# Adherence to Hygiene Protocols and Doxycycline Therapy in Ameliorating Lymphatic Filariasis Morbidity in an Endemic Area Post-Interruption of Disease Transmission in Ghana

**DOI:** 10.4269/ajtmh.24-0313

**Published:** 2024-10-01

**Authors:** Linda Batsa Debrah, Ute Klarmann-Schulz, Jubin Osei-Mensah, Janina M. Kuehlwein, Yusif Mubarik, Jennifer Nadal, Nana Kwame Ayisi-Boateng, Arcangelo Ricchiuto, Vera Serwaa Opoku, Sarah M. Sullivan, Derrick Adu Mensah, John Horton, Abu Abudu Rahamani, Philip J. Budge, Stephen Gbedema, Patricia Jebett Korir, John Opoku, Kenneth Pfarr, Derrick Boateng Kontoh, Angelika Kellings, Charles Gyasi, Michael Agyemang Obeng, Barbara Gruetzmacher, Fatima Amponsah Fordjour, Inge Kroidl, Sacha Horn, Eunice Kyaakyile Kuutiero, Caroline Wauschkuhn, Abdallah Ngenya, Charles Mackenzie, Samuel Wanji, Akili Kalinga, Eric A. Ottesen, Achim Hoerauf, Alexander Yaw Debrah

**Affiliations:** ^1^ Kumasi Centre for Collaborative Research in Tropical Medicine (KCCR), Kwame Nkrumah University of Science and Technology (KNUST), Kumasi, Ghana;; ^2^ Department of Clinical Microbiology, Kwame Nkrumah University of Science and Technology (KNUST), Kumasi, Ghana;; ^3^ German-West African Center for Global Health and Pandemic Prevention (G-WAC), partner site Kumasi, Ghana;; ^4^ Institute for Medical Microbiology, Immunology and Parasitology (IMMIP), University Hospital Bonn, Bonn, Germany;; ^5^ German Center for Infection Research (DZIF), partner site Bonn-Cologne, Germany;; ^6^ Institute for Medical Biometry, Informatics and Epidemiology (IMBIE), University Hospital Bonn, Bonn, Germany;; ^7^ School of Veterinary Medicine, Kwame Nkrumah University of Science and Technology (KNUST), Kumasi, Ghana;; ^8^ Department of Medicine, School of Medicine and Dentistry, Kwame Nkrumah University of Science and Technology (KNUST), Kumasi, Ghana;; ^9^ Neglected Tropical Diseases Support Center, Task Force for Global Health, Decatur, Georgia;; ^10^ Tropical Projects, Hitchin, United Kingdom;; ^11^ Washington University School of Medicine, St. Louis, Missouri;; ^12^ Department of Pharmaceutics, Faculty of Pharmacy and Pharmaceutical Sciences, College of Health Sciences, Kwame Nkrumah University of Science and Technology (KNUST), Kumasi, Ghana;; ^13^ Clinical Study Core Unit Bonn (SZB), Institute of Clinical Chemistry and Clinical Pharmacology, University Bonn, Bonn, Germany;; ^14^ Department of Microbiology, University for Development Studies (UDS), Tamale, Ghana;; ^15^ Division of Infectious Diseases and Tropical Medicine, Medical Center of the University of Munich (LMU), Munich, Germany;; ^16^ German Center for Infection Research (DZIF), partner site Munich, Germany;; ^17^ National Institute for Medical Research (NIMR), Dar es Salaam, Tanzania;; ^18^ Department of Microbiology and Parasitology, University of Buea, Buea, Cameroon;; ^19^ German-West African Center for Global Health and Pandemic Prevention (G-WAC), partner site Bonn, Germany;; ^20^ Faculty of Allied Health Sciences, Kwame Nkrumah University of Science and Technology (KNUST), Kumasi, Ghana

## Abstract

Filarial lymphedema (LE) remains a significant global problem despite the progress made toward elimination of lymphatic filariasis (LF). In Ghana, the main approach to LF is preventive chemotherapy, but this has minimal impact on individuals who have already developed LE. In 2018–2020, a 24-month randomized, double-blind, placebo-controlled trial was conducted to evaluate the efficacy of stringent hygiene measures using the Essential Package of Care with or without additional administration of doxycycline (DOX) to improve filarial leg LE. This study enrolled 356 participants with LE stages 1–3 from two districts in the Upper East Region of Ghana. In addition to regular training on appropriate care for their affected legs, participants were randomized to receive 6 weeks of either 200 mg/day DOX (*n* = 117), 100 mg/day DOX (*n* = 120), or matching placebo (*n* = 119). Participants were seen every 2 months, with clinical measurements done at 6, 12, 18, and 24 months to assess the status of affected legs. There was a trend toward later appearance of acute attacks after DOX, but surprisingly, DOX showed no effect on LE stage progression. In all groups, leg LE improvement was more common (DOX 200 mg: *n* = 23 [20%]; DOX 100 mg: *n* = 23 [19.5%]; placebo: *n* = 32 [27.4%]) than LE worsening (DOX 200 mg: *n* = 2 [1.7%]; DOX 100 mg: *n* = 3 [2.5%]; placebo: *n* = 2 [1.7%]). Overall, these data show a strong benefit from adherence to a strict hygiene protocol, with some added potential benefit for DOX in preventing acute attacks.

## INTRODUCTION

Lymphatic filariasis (LF), a mosquito-borne parasitic disease, is known to cause permanent disability and deformity through impairment in the lymphatic system and subsequent abnormal enlargement of body parts such as limbs, scrotum, breast, and vulva.^1^ As one of the neglected tropical diseases, LF is targeted for elimination by the WHO through the Global Program for Elimination of Lymphatic filariasis (GPELF) by 2030.^2^ The Essential Package of Care developed toward the elimination process consists of two pillars: 1) eliminating transmission of LF through mass drug administration (MDA) and 2) alleviating the suffering of individuals with chronic conditions such as lymphedema (LE; enlargement of other body parts) and hydrocele (enlargement of the scrotum) through morbidity management and disability prevention (MMDP) methods. Mass drug administration treatment uses albendazole (ALB) and ivermectin (IVM) in areas with onchocerciasis; diethylcarbamazine (DEC), IVM, and ALB in areas without onchocerciasis; and ALB only in areas where loa is coendemic.^3^ Triple therapy of IVM, ALB, and DEC has also been found to be safe and efficacious by having better macrofilaricidal activity compared with either IVM + ALB or DEC + ALB alone.^4^ Morbidity management and disability prevention includes guidance in applying simple measures such as foot hygiene, elevation, and exercise to manage LE to prevent its progression to elephantiasis and debilitating, inflammatory episodes of acute adenolymphangitis (ADL). Surgery is recommended to repair hydrocele.

Through the MDA program, LF prevalence declined from 199 million in 2000 to 51 million (by about 74%) as of 2018.^3^^,^^5^ However, there was only a marginal reduction in chronic cases from about 40 million people in 2000 to about 36 million people in 2014, even with the WHO recommendation of MMDP.^1^^,^^6^ As a result, countries that have presently achieved elimination will still have many individuals with chronic manifestations for years to come.

In Ghana, MDA started in 2000 through the Ghana Filariasis Elimination Program, and a significant achievement has been recorded in terms of interruption in transmission. Morbidity management and disability prevention programs started when the WHO rolled out a program for managing chronic conditions. In 2002, surgeons were trained on optimizing surgical hydrocelectomies, and the Ghana Health Service (GHS) developed a manual for LE management.^7^^,^^8^ The scaling up of MMDP has been slow and has not received maximum attention.

In addressing MMDP issues from the southern part of Ghana, where transmission was ongoing^9^ and hygiene was not strictly adhered to, Mand et al.^10^ undertook a study in 2012 using 200 mg/day of DOX for 6 weeks on top of the standard MDA program and hygiene management. They saw a significant improvement in LE manifested as reduction in LE stage (staging according to Dreyer et al.^11^) compared with individuals who received amoxicillin or standard MDA and hygiene management alone, as well as a reduction in the number of participants having ADL attacks.^10^ Doxycycline depletes *Wolbachia* endobacteria required by filariae, killing both microfilaria and adult worms; DOX also reduces inflammatory and angiogenic growth factors associated with chronic disease.^12^^,^^13^ A DOX dose of 100 mg/day for 6 weeks is effective for depletion of *Wolbachia* in *Onchocerca volvulus*.^14^ Therefore, in this study in addition to the 200 mg/day of DOX, the activity of 100 mg/day of DOX for MMDP was also investigated. The aim of the study was to assess the impact of two different dosages of DOX (100 and 200 mg/day) along with strict adherence to hygiene measures in the management of LF disease.

## MATERIALS AND METHODS

### Trial area description.

This trial was carried out in 125 mainly rural communities in 13 subdistricts in the Kassena Nankana East Municipal (KNEM) and Kassena Nankana West (KNW) District of the Upper East Region of Ghana, which were previously mapped as being endemic for LF and very recently considered as LF hot spot districts (Supplemental Tables 1 and 2).^9^^,^^15^^,^^16^ Two other subdistricts, Katiu-Nankong and Kayoro, were excluded because of LF co-endemicity with onchocerciasis, another filarial disease. The two districts (KNEM and KNW) are geographically located in the guinea savanna woodland area, with an average annual rainfall of approximately 1,000 mm, most of which falls between May and September.^17^ The districts show a typically Sahelian ecological setting with temperatures typically ranging between 16°C and 41°C throughout the year.^17^ Communities within these districts are mainly rural with dispersed settlements of extended family compounds surrounded by farmlands.^16^ Numerous small dams exist in the districts, providing favorable breeding sites for mosquitoes, which facilitates transmission of LF infection. Mass drug administration with IVM and ALB had been stopped in the KNEM district as part of pre-transmission assessment surveys (pre-TASs) in 2015. However, in 2018, MDA resumed in the KNEM district and continued through the period of the trial (in 2018 and 2019), except in 2020 when MDA was not done because of the COVID-19 pandemic. In communities in the KNW district, MDA was not done during the entire period of the trial, although no pre-TAS activities were planned to take place, and it remains an LF hot spot.

### Recruitment of trial participants.

Recruitment was carried out directly in the communities. A few days prior to recruitment, announcements were made via community information centers or by a “gong-gong” beater (a local means of giving information to community residents in which the “beater” moves around sounding a metallic instrument known as the gong-gong, while intermittently shouting out the information). All community residents, including the elders and opinion leaders, were informed by community health volunteers (CHVs) to gather at chosen social centers through these announcements. The study was explained in English and then in the local languages, Kasem and Nankam, at the meeting on the planned date of recruitment start.^17^ All interested individuals between 14 and 65 years of age who intended to participate in the trial signed the informed consent form for screening prior to initial screening and selection. During screening, physical examinations were conducted to assess the physical health of the volunteers; questionnaires regarding previous MDA intake and ADL episodes, medical history, and concomitant medications were undertaken; LE staging was done; and blood was taken for hematological, parasitological, and biochemistry analysis. To prevent MDA treatment round and ADL episode recall bias, responses from participants were cross-checked with the records kept by CHVs/community drug distributors for every household in the last 15–20 years of MDA treatment. This was done before the numbers were used for analysis.^16^ All eligible participants were invited to come to the enrollment visit, which was also done in the trial communities. During the enrollment visit, LE-specific examinations were carried out. After the enrollment visit, participants who were eligible for the trial were randomized into three different treatment arms. Participants who failed the screening procedure were encouraged to take part in the regular MDA “standard of care” and to adhere to the GPELF Management Guidelines.^18^

### Study population and eligibility criteria.

Only participants with LE of the lower limbs were included in the study. Aside from the trial protocol-specific screening procedures described previously,^16^ physical and medical examinations were conducted by the trial clinicians to assess the fitness of potential trial participants for inclusion in the trial. Included in this trial were men or nonpregnant women between 14 and 65 years of age with LE in at least one leg of grade 1–6 measured on the 7-point Dreyer scale (an LE staging/grading system that is generally accepted and widely used among filarial experts),^11^^,^^19^ a body weight of at least 40 kg, and residence in an LF-endemic area for at least 2 years. Participants had to be able and willing to give informed consent and have the capacity to apply established standardized methods of hygiene. Women of childbearing age were educated on the use of an approved and effective method of contraception (including abstinence) before, during, and for at least 2 weeks after administration of the study drugs. Individuals were excluded from participation if they had clinical or biological evidence of hepatic or renal dysfunction, disease of the central nervous system, or evidence of severe comorbidities aside from features of filarial disease. Potential participants with signs or histories of alcohol or drug abuse; adverse reactions to DOX or other tetracyclines; photosensitivity reactions after taking drugs or concomitant medication with antacids containing aluminum, magnesium, or sucralfate, diuretics or sulfonylurea, coumarin, or antibiotics other than DOX; and who were not able to discontinue these medications were also excluded from the trial. Exclusion criteria also included hemoglobin levels less than 8 g/dL; neutrophil counts less than 1,100/mm^3^; platelet counts less than 100,000/mm^3^; creatinine, aspartate transaminase (AST), glutamic oxaloacetic transaminase (GOT), alanine transaminase (ALT), glutamic pyruvic transaminase (GPT), and γ-glutamyl transaminase (γ-GT) levels more than two times the upper limit of normal; and in the case of women, a positive urine pregnancy test. In addition, participants with any significant condition (including medical and psychological/psychiatric disorders) that, in the opinion of the study investigator, might interfere with the conduct of the study, were also excluded from the trial.

### Sex distribution.

No sex ratio was stipulated in the trial protocol because the results of previous preclinical and clinical studies did not indicate any sex-specific effects of the trial treatment in terms of efficacy and safety.^10^^,^^12^^,^^20^^–^^22^

### Trial design.

This was a prospective, multinational (involving four other countries: Tanzania, Mali, Sri Lanka, and India), randomized, placebo-controlled, double-blind, parallel-group interventional trial in which all enrolled participants received morbidity management and hygiene training as defined in the GPELF Management Guidelines in addition to daily DOX or placebo matching the DOX tablets for 6 weeks.^16^^,^^18^ To simplify treatment in rural settings, active DOX and placebo were similarly packaged in blister treatment packs.^16^

**Figure 1.**
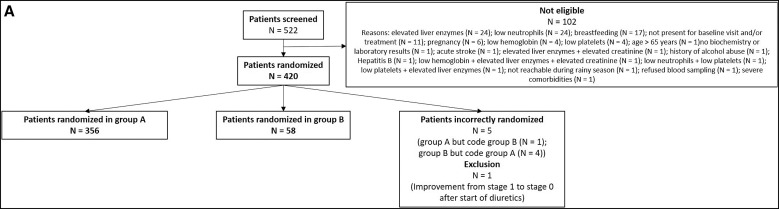

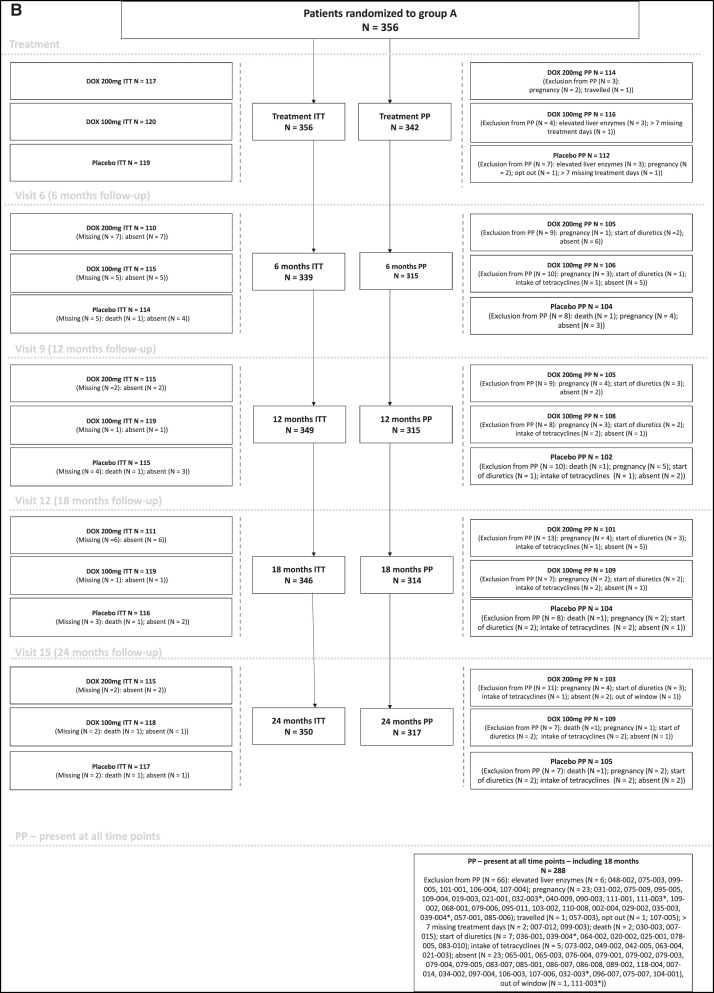
(**A**) Flowchart – recruitment and randomization. From the 420 eligible participants, 356 were randomized in group A (LE stages 1–3) and 58 in group B (LE stages 4–6). Five participants were incorrectly randomized. One participant belonged to group A but was randomized in group B, and four participants were randomized in group A but belonged to group B. This became apparent only after treatment was carried out. Another participant started with diuretic treatment right after trial treatment. This resulted in improvement from stage 1 to stage 0. It was decided in consultation with the Data Safety and Monitoring Board before unblinding of the study to exclude the data of these six participants from all but the safety analyses. (**B**) Flowchart (see next page) – treatment allocation. The number of participants in group A were randomized to one of the three treatment groups; their presence/absence during the follow-up visits as well as their belonging to the ITT and/or PP analysis sets for the respective time points and the reason for their absence or exclusion from PP analysis are shown. ITT = intention-to-treat; PP = per-protocol.

All enrolled trial participants had clinically evaluable LE of the leg(s)^16^ and were grouped into two cohorts based on LE stage (Figure 1A): the first (group A) included 356 participants with LE stages 1–3, and the second (group B) comprised 58 participants with LE stages 4–6, which was independently assessed in the form of a nonconfirmatory pilot trial because previous controlled trials by our group had revealed the efficacy of DOX 200 for 6 weeks in ameliorating lower filarial LE stages (1–3)^10^^,^^12^; however, there were not yet sufficient results for DOX 200 for 6 weeks in individuals with higher LE stages (4–6).

Group A participants were randomized to receive either 200 mg/day DOX (DOX 200: *n =* 117) once daily, 100 mg/day DOX (DOX 100: *n =* 120) once daily, or placebo-matching DOX (*n =* 119; Figure 1B). Group B participants were randomized to receive either 200 mg/day DOX (DOX 200: *n =* 29) once daily or placebo-matching DOX (*n =* 29).^16^ In this paper, only the results for group A are described and discussed; the results for group B (nonconfirmatory pilot trial) will be published separately.

Treatment was directly observed (DOT), with daily tablet intake supervised by a trial clinician. Because 200-mg DOX is not recommended for patients below 50 kg in weight, participants who were randomized to receiving 200 mg/day DOX but had a body weight <50 kg had their dose reduced to 100 mg/day; one set of tablets within a single blister bed was removed prior to their treatment for the 6-week period as previously described.^16^

To assess the occurrence of acute attacks, follow-ups were undertaken every 2 months until 24 months after treatment onset. Major follow-ups within the study period were scheduled at 6, 12, 18, and 24 months after treatment onset to repeat the baseline procedures/measurements and assess adherence to hygiene measures and to reinforce teaching of limb care practices.

### Justification for clinical trial sample size.

The trial sample size was based on the outcome from a previous study by our group,^10^ in which a progression of approximately 5% was seen in the 200 mg/day DOX group after 2 years, whereas the placebo group had a progression of about 56%. To account for additional emphasis on hygiene measures in all groups in the present trial, which was expected to reduce progression in all treatment arms, the progression for the placebo group was modestly estimated to be 25% (less than half of the over 50% observed in the previous study). The sample size was calculated based on the above assumptions with a power of 95% for the DOX 200-mg group and 81% for the DOX 100-mg group, respectively, to show superiority to the placebo group with the inclusion of 84 participants per treatment arm. A drop-out rate of 30% was estimated, leading to a final sample size of 120 participants per treatment arm and 360 participants in total.

### Selection of filarial LE trial participants.

Individuals with swelling of the lower limb(s) or leg(s) not due to trauma who had experienced at least one episode of acute attacks known as *acute ADL* were identified as filarial LE cases. Lymphedema-affected participants were selected based on history and other physical examination procedures. Participants with edema of the lower limbs in the filarial-endemic communities were asked questions on the etiology of their condition, previous experiences of ADL attacks precipitated by the swelling of the lymph nodes, feverishness, swelling of the legs, and peeling off of the skin at the recession of the ADL attack.

Participants had their LE-affected legs staged from 1–7 using the Dreyer LE staging scale.^10^^,^^11^ Although there have been numerous international attempts to classify and stage LE, no single method of staging has provided a comprehensive view of the disease by defining or describing its etiopathophysiology and the significant overlap between recorded stages.^23^ Thus, the process of LE classification and staging has been branded deficient because the definitive features on the affected limb used for staging and grading by different methods differ and vary widely.^23^ However, among the four main LE staging methods/systems used in categorizing affected limbs (International Society of Lymphology/International Lymphedema Framework, WHO, Staging by Dreyer et al.,^11^ and staging described for podoconiosis), the Dreyer’s system appears to give a much more detailed account of LE progression.^24^ One study also reported a high level of consistency among LE staging assessors who were health workers using the Dreyers’ 7-stage classification compared with the other staging criteria.^25^ Thus, this study relied on Dreyer’s 7-stage system as it is generally accepted and is the most widely used staging system among the filarial community of experts. The LE staging was done before enrollment, randomization, and treatment and at the major follow-up time points. In accordance with Dreyer staging,^11^ when two or more features corresponding to different LE stages were present on one leg, that leg was classified with the feature corresponding to the highest stage. Male participants were further asked if they had any visible scrotal swelling and were then clinically examined. A study clinician was responsible for the identification and confirmation of hydrocele cases.

### Randomization.

To prevent bias in the trial design, randomization lists were generated by the manufacturer of the study drugs (Piramal, Morpeth, United Kingdom) using block randomization.^16^ Patients with LE stages 1–3 (group A: *n =* 360) and LE stages 4–6 (group B: *n =* 60) were randomized separately.

### Blinding.

Trial participants, care providers, and outcome assessors were blinded to the trial drugs received by the participants. This was achieved by sequentially entering participants when they were enrolled into the study. Thus, the first participant enrolled received drug pack 1, the second participant received drug pack 2 in this order, from one community to the other.

### Laboratory examinations.

Blood samples were collected at baseline for circulating filarial antigen (CFA) testing and quantification of microfilariae (MF) as described in a previous study,^26^ as well as for hematological analysis, biochemistry analysis, and ELISA tests. Similarly, during the major follow-up periods at 6, 12, and 24 months after treatment, blood samples were taken from each participant to undertake various laboratory investigations as described above. The CFA tests were done using the Alere Filariasis Test Strips (Alere Scarborough, Inc., Scarborough, ME). Sedgewick and Giemsa/Filter MF counts were done once at baseline after collection of night blood samples for participants who were filariasis test strip/CFA positive, and the number of MF counted was recorded as MF per milliliter.^27^

When taken in high doses for prolonged periods, DOX has been reported to be toxic to the functioning of the kidneys and liver in dogs.^28^ Therefore, to ensure trial participant safety, clinical chemistry tests were done to assess the state of a potential trial participant’s kidneys and liver prior to enrollment. Creatinine, AST, ALT, and γ-GT were the analytes/parameters selected to be measured to ascertain kidney and liver function status of trial participants for enrollment. All participants who had analyte results above the cutoff levels per the trial protocol were not enrolled in the trial and were rather advised to take the annual MDA. Clinical laboratory tests were repeated after 21 days of treatment and at the end of the 6-week treatment. Individuals with abnormal results after 21 days had treatment stopped.

The hematological parameters of potential trial participants assessed were platelet counts, neutrophil counts, and hemoglobin levels. Participants with abnormal levels of platelet counts, neutrophils counts, or hemoglobin levels were not enrolled in the trial.

Urine pregnancy tests were done for all females below 55 years of age during screening, at baseline, prior to the first treatment, and after 14, 28, and 42 days of treatment as well as at the 2-, 6-, 12-, and 24-month follow-up time points. The HCG accurate pregnancy test kit (Registration number FDB/D16-11170; Guangzhou Wondfo Biotech Company Limited, Guangzhou, China) was used to perform these tests at all time points.

### Field examinations.

#### Clinical photography.

Digital clinical photographs of both legs of trial participants were taken and stored in a digital format at baseline and at the follow-up time points of 6, 12, 18, and 24 months. The distance, lighting, and background were standardized for each participant, and all efforts were made to ensure comparability.

#### Training for care and hygiene of affected limbs.

All patients were initiated into a program of cleaning of the affected limbs based on the principles outlined in the booklet *New Hope* for persons with LE.^11^ Prior to enrollment, all participants were trained to use a standardized protocol of leg care hygiene and management that involved washing the limb(s) and using a diary for recording ADL attacks. Lower limb hygiene care and management training involved the following steps: cleaning of the affected limb(s) daily with soap and water; keeping the affected limb(s) dry; clipping the nails; applying topical antibacterial and antifungal creams to open sores, toe webs, nails, and sides of the feet every night; regular elevation of the affected limb when in a resting position; limb exercises as instructed; and encouraging and monitoring the use of appropriate footwear. Community health volunteers were engaged to assist persons who could not read and write to record the ADL attacks in the diary, as done in a previous study.^10^ Trial participants were visited every 2 months and were actively encouraged to continue with the hygiene protocol for the entire duration of the trial. Refresher trainings on the protocol were offered to all trial participants at 4, 6, 12, 18, and 24 months after treatment—the same time points at which participants were assessed for leg care and hygiene. Each participant also received soaps, towels, and plastic bowls at regular intervals during the trial to facilitate adherence to the leg hygiene protocol.

#### Quality of life assessment.

The 12-item WHO Disability Assessment Schedule (WHODAS 2.0) questionnaire^29^ was used to assess the disability and thus the quality of life (QoL) of trial participants at baseline and at 12 and 24 months. The WHODAS tool measures an individual’s level of functioning in six major life domains: 1) cognition (understanding and communication); 2) mobility (ability to move and get around); 3) self-care (ability to attend to personal hygiene, dressing, and eating and to live alone); 4) getting along (ability to interact with other people); 5) life activities (ability to carry out responsibilities at home, work, and school); and 6) participation in society (ability to engage in community, civil, and recreational activities). The questionnaire was administered using translations in the local language by trained personnel. Because this questionnaire assesses self-perceived disability, a higher score indicates more disability (lower QoL).

#### Circumference and volume measurements of legs.

To measure the limb volume and circumference of trial participants with LE, two methods were used: a portable three-dimensional infrared imaging tool known as the Lymphatech^®^ scanner (Atlanta, GA)^16^^,^^30^ and a traditional tape measure tool.^10^ Duplicate measurements of the volume of the lower legs were taken for each leg below the knee with the Lymphatech scanner. Using the tape measure tool, duplicate leg circumference measurements were taken for each leg at 10 cm posterior to the tip of the large toe and at 12, 20, and 30 cm from the sole of the foot.^10^ Measurements using both methods were done at baseline and 6, 12, and 24 months after treatment start.

### Assessment of safety.

Adverse events (AEs) were assessed for a period of 4 months after DOT onset. Assessments involved 1) occurrence of an AE; 2) intensity of the AE (grade 0 [none], grade 1 [mild], grade 2 [moderate], grade 3 [severe]); 3) occurrence of a serious adverse event (SAE); 4) relation to treatment (definite, probable, possible, remote, not related); 5) outcome of the AE (restored, improved, unchanged, deteriorated, death, unknown, overcome with sequelae); and 6) intervention provided. The SAEs that occurred during the whole study period of 24 months were reported to the regulatory authority (Ghana Health Service Ethics Review Committee [GHS-ERC] and Kwame Nkrumah University of Science and Technology [KNUST]–Committee on Human Research Publication and Ethics [CHRPE]) within 48 hours after awareness by the research team. The AEs and SAEs were coded using MedDRA v. 23.1.

## STATISTICAL ANALYSES

All trial data were entered in REDCap^®^ (Research Electronic Data Capture, San Francisco, CA)^31^^,^^32^ during the trial using double data entry on-site in Ghana. REDCap was hosted at the Institute for Medical Biometry, Informatics and Epidemiology of the University Hospital Bonn, Germany.

Before de-blinding of the trial, three different datasets were established to analyze the data: 1) the Safety (SAF) set, which included all participants randomized (*n =* 420); 2) the intention-to-treat (ITT) set, which included all eligible participants correctly randomized (*n =* 414); and 3) the per-protocol (PP) set(s), which included all eligible participants who completed the treatment per protocol and were present at the respective time points without any medical condition that would have excluded them from analysis (see Figure 1B). The SAF set was used for the analysis of the safety data only. The ITT set was used for all analyses, and the PP sets were used to confirm the univariate and bivariate ITT analyses.

Rules were established prior to the start of the study as to which leg would be evaluated if two legs were affected. For one leg with stage 1–3 and one with stage 4–6, the lower stage was evaluated; for both legs with stage 1–3, the leg with the higher stage was chosen. In the event that both legs had the same stage, computerized randomization during unblinding was used to decide which study leg was evaluated.

The statistical analyses were performed using SAS Version 9.4 (SAS Institute Inc., Cary, NC). Descriptive statistics of continuous variables were presented as mean ±SEM (SEM) for normally distributed variables and median (interquartile range [IQR]) for non-normally distributed variables. Categorical variables were presented as numbers and percentages. For continuous variables, an ANOVA or Kruskal-Wallis test was used to show differences between treatment groups at baseline. For all categorical variables, the exact Fisher’s test was used, when possible, to assess treatment differences. The statistical significance was defined as *P <*0.05.

Mixed-effects models with binary outcomes for progression, improvement, and hygiene status were used (Procedure for Generalized Linear Models in Statistical Analysis System). Effects are presented as odds ratios (ORs) with 95% CIs. We used a linear mixed-effects model with WHODAS 2.0 as the outcome and presented the estimate (β) with 95% CI. Predictor variables for the mixed-effects models included the following baseline variables: sex (male/female), age, weight, years in endemic area, other leg affected, treatment (DOX 200/DOX 100/placebo), and region (Kassena Nankana East and Kassena Nankana West) as well as the following time-dependent covariables (taking changes during the follow-up period into account): LE staging (stage 1, 2, or 3), LE change (no change/progression/improvement), FTS positivity (active LF infection), hygiene status (limb not clean/limb clean), ADL attack during the previous 6 months (no/yes), and season at time of assessment (dry/rainy season). For analysis of time to first occurrence of ADL, we plotted the Kaplan-Maier curve and used the log-rank test to show a difference between treatments. To represent the occurrence of all ADLs, we used the approach of Anderson and Gil,^33^^,^^34^ which generates a Cox model formulated in terms of increments in the number of events along the time line; these effects are presented as hazard ratios (HRs) with 95% CIs.

## RESULTS

Out of 522 volunteers screened, 420 were eligible for enrollment in the trial and were randomized to either group A (stages 1–3) or group B (stages 4–6) as shown in Figure 1A. Results for group A will be described and discussed here; the results for group B (nonconfirmatory pilot trial) will be presented in a separate publication.

Of the 356 participants randomized in group A, 117 received DOX 200 mg for 6 weeks, 120 received DOX 100 mg for 6 weeks, and 119 received placebo (Figure 1B). After enrollment and randomization, the first participants received the first dose of trial medication on May 18, 2018.

In total, 342 participants (96.1%) completed the trial per protocol. Twelve participants (3.4%) did not complete 42 days of treatment (DOX 200: *n =* 3, DOX 100: *n =* 4, placebo: *n =* 7) because of pregnancy (*n =* 4), elevated liver enzymes (*n =* 6), traveling (*n =* 1), and an active decision to drop out (*n =* 1). In addition, two participants completed the full 42 days of treatment but missed more than 7 days of treatment in between the 42 days. This led to the exclusion of the 14 participants from the PP analysis set. Of the 342 participants who completed the 42 days of treatment, 66 participants missed 1 treatment day, 30 missed 2, 13 missed 3, four missed 4, two missed 5, and two missed 6 treatment days in between, but all of them for less than 4 consecutive days. All the missed doses were administered during additional treatment days at the end of the treatment period. Overall, 225 (74.6%) participants took the drugs for 42 consecutive days.

The last participant was followed up after 24 months on August 20, 2020.

### Baseline data.

Baseline characteristics were similar between the three treatment groups, except for slight imbalances in sex (more men [20%] in the placebo group than in the other two groups [11.1% and 7.5%; *P =* 0.0071]), and the proportion of study participants with weight above or below 50 kg. There were more participants weighing ≤50 kg in the DOX 200 group (*n =* 17[14.5%]) than in the other two groups (*n =* 6 [5%] and *n =* 10 [8.4%]; *P =* 0.039). Marital status also varied between groups (*P =* 0.0264; Supplemental Table 3).

Table 1 and Supplemental Table 3 show the trial-specific variables directly. Overall, there were no differences between the treatment groups regarding LE characteristics, ADL history, hygiene assessment, and the quality of life at baseline. Regarding the study legs, there was a trend toward a higher proportion of participants with stage 3 disease in the placebo group (*P =* 0.0817).

**Table 1:** Trial-Specific Variables

Baseline Data: Demographics							
Variable			DOX 200 mg	DOX 100 mg	Placebo	Total	*P*-Value
Sex	Female	*N* (%)	104 (88.9)	111 (92.5)	94 (79)	309 (86.8)	**0.0071** *
	Male	*N* (%)	13 (11.1)	9 (7.5)	25 (21)	47 (13.2)	
Age (years)		*N*	117	120	119	356	0.3067^†^
		Mean ± SEM	47.6 ± 0.9	47.1 ± 0.9	45.8 ± 0.8	46.8 ± 0.5	
		95% CI Mean	[45.9–49.3]	[45.4–48.9]	[44.2–47.4]	[45.9–47.8]	
		Min–max	26–65	17–65	16–65	16–65	
Districts	Kassena Nankana East Municipal	*N* (%)	48 (41)	50 (41.7)	51 (42.9)	149 (41.9)	0.9628*
	Kassena Nankana West	*N* (%)	69 (59)	70 (58.3)	68 (57.1)	207 (58.1)	
Body Weight		*N*	117	120	119	356	0.4915^†^
		Mean ± SEM	63.2 ± 1.2	64.8 ± 1.2	63.2 ± 0.9	63.7 ± 0.6	
		95% CI Mean	[60.9–65.5]	[62.5–67.1]	[61.3–65]	[62.5–65]	
		Min–max	43–112	42–120	43–100	42–120	
Weight in Categories	≤50 kg	*N* (%)	17 (14.5)	6 (5)	10 (8.4)	33 (9.3)	**0.039** *
	>50 kg	*N* (%)	100 (85.5)	114 (95)	109 (91.6)	323 (90.7)	
Duration Stayed in Endemic Area (years)		*N*	117	120	119	356	0.3795^†^
		Mean ± SEM	44.9 ± 1.1	46.5 ± 0.9	44.7 ± 0.9	45.4 ± 0.6	
		95% CI Mean	[42.7–47.1]	[44.6–48.3]	[42.8–46.5]	[44.2–46.5]	
		Min–max	2–65	15–65	2–65	2–65	
Previous MDA Rounds		*N*	68	76	76	220	0.9093^‡^
		Median, IQR	4; 4	4; 3	4.5; 3	4; 3	
		95% CI Median	[4–5]	[4–5]	[4–5]	[4–5]	
		Min–max	1–20	1–20	1–17	1–20	
Antigenemia (filarial test strip)	Negative	*N* (%)	89 (76.1)	100 (83.3)	92 (77.3)	281 (78.9)	0.3336*
	Positive	*N* (%)	28 (23.9)	20 (16.7)	27 (22.7)	75 (21.1)	
Microfilariae (MF)	Negative	*N* (%)	28 (100)	19 (95)	27 (100)	74 (98.7)	0.2667*
	Positive	*N* (%)	0 (0)	1 (5)	0 (0)	1 (1.3)	

Baseline Data: Participants’ Characteristics							
Variable			DOX 200 mg	DOX 100 mg	Placebo	Total	*P*-Value
Duration of LE (years)	*N*		96	97	95	288	0.2627^‡^
	Median, IQR		10; 18.5	10; 15	10; 20	10; 15	
	95% CI Median		[9; 17]	[8; 12]	[10; 16]	[10; 10]	
	Min–max		1–55	1–40	1–44	1–55	
Number of Legs Affected	Only One Leg	*N* (%)	91 (77.8)	93 (77.5)	98 (82.4)	282 (79.2)	0.5955*
	Both Legs	*N* (%)	26 (22.2)	27 (22.5)	21 (17.6)	74 (20.8)	
Both Legs Have the Same Stage	No	*N* (%)	9 (34.6)	11 (40.7)	8 (38.1)	28 (37.8)	0.9538*
	Yes	*N* (%)	17 (65.4)	16 (59.3)	13 (61.9)	46 (62.2)	
LE Staging Study Leg	1: Swelling Is Reversible	*N* (%)	1 (0.9)	2 (1.7)	2 (1.7)	5 (1.4)	0.0817*
	2: Swelling Is Not Reversible	*N* (%)	72 (61.5)	79 (65.8)	59 (49.6)	210 (59)	
	3:Presence Of Shallow Skinfolds	*N* (%)	44 (37.6)	39 (32.5)	58 (48.7)	141 (39.6)	
ADL Attacks Since (years)	Unknown^‖^	*N*	29	31	31	91	0.845^§^
	<1 Year	*N* (%)	1 (1.1)	1 (1.1)	2 (2.3)	4 (1.5)	
	≥1–5 Years	*N* (%)	17 (19.3)	23 (25.8)	17 (19.3)	57 (21.5)	
	≥5–10 Years	*N* (%)	19 (21.6)	15 (16.9)	14 (15.9)	48 (18.1)	
	≥10–15 Years	*N* (%)	14 (15.9)	18 (20.2)	20 (22.7)	52 (19.6)	
	≥15–20 Years	*N* (%)	4 (4.5)	7 (7.9)	6 (6.8)	17 (6.4)	
	≥20 Years	*N* (%)	33 (37.5)	25 (28.1)	29 (33)	87 (32.8)	
Duration of Last ADL Attack (days)	N		114	119	118	351	0.6318^‡^
	Median, IQR		7; 4	7; 6	7; 4	7; 4	
	95% CI Median		[5–7]	[7–7]	[5–7]	[7–7]	
	Min–max		0–90	0–30	1–90	0–90	
Number of Attacks Within the Last Year (only patients who had attacks)	*N*		82	83	95	260	0.2404^‡^
	Median, IQR		1; 1	1; 1	1; 1	1; 1	
	95% CI Median		[1–2]	[1–2]	[1–1]	[1–1]	
	Min–max		1–6	1–6	1–9	1–9	
Overall Hygiene—Limb Washed and Clean	Missing	*N*	1	0	0	1	0.4849*
	No	*N* (%)	21 (18.1)	15 (12.5)	19 (16)	55 (15.5)	
	Yes	*N* (%)	95 (81.9)	105 (87.5)	100 (84)	300 (84.5)	
WHODAS 2.0 Score	N		117	120	119	356	0.8029
	Mean ± SEM		28.7 ± 1.8	27.5 ± 1.7	27.1 ± 1.6	27.8 ± 1.0	
	95% CI Mean		[25–32.3]	[24.1–30.9]	[24–30.3]	[25.8–29.7]	
	Min–max		0–100	0–83.4	0–68.8	0–100	

ADL = adenolymphangitis; ANOVA = analysis of variance; DOX = doxycycline; IQR = interquartile range; LE = lymphedema; MDA = mass drug administration; Min-Max = minimum to maximum; SEM = standard error of the mean; WHODAS = WHO Disability Assessment Schedule. Boldface font indicates variables showing significant *P*-values (significant differences).

* Fisher’s exact test.

^†^ ANOVA.

^‡^ Kruskal-Wallis test.

^§^ χ^2^ test.

^‖^ Participant could not recall the last time he/she had an attack.

### Lymphedema progression and improvement.

**Figure 2.**
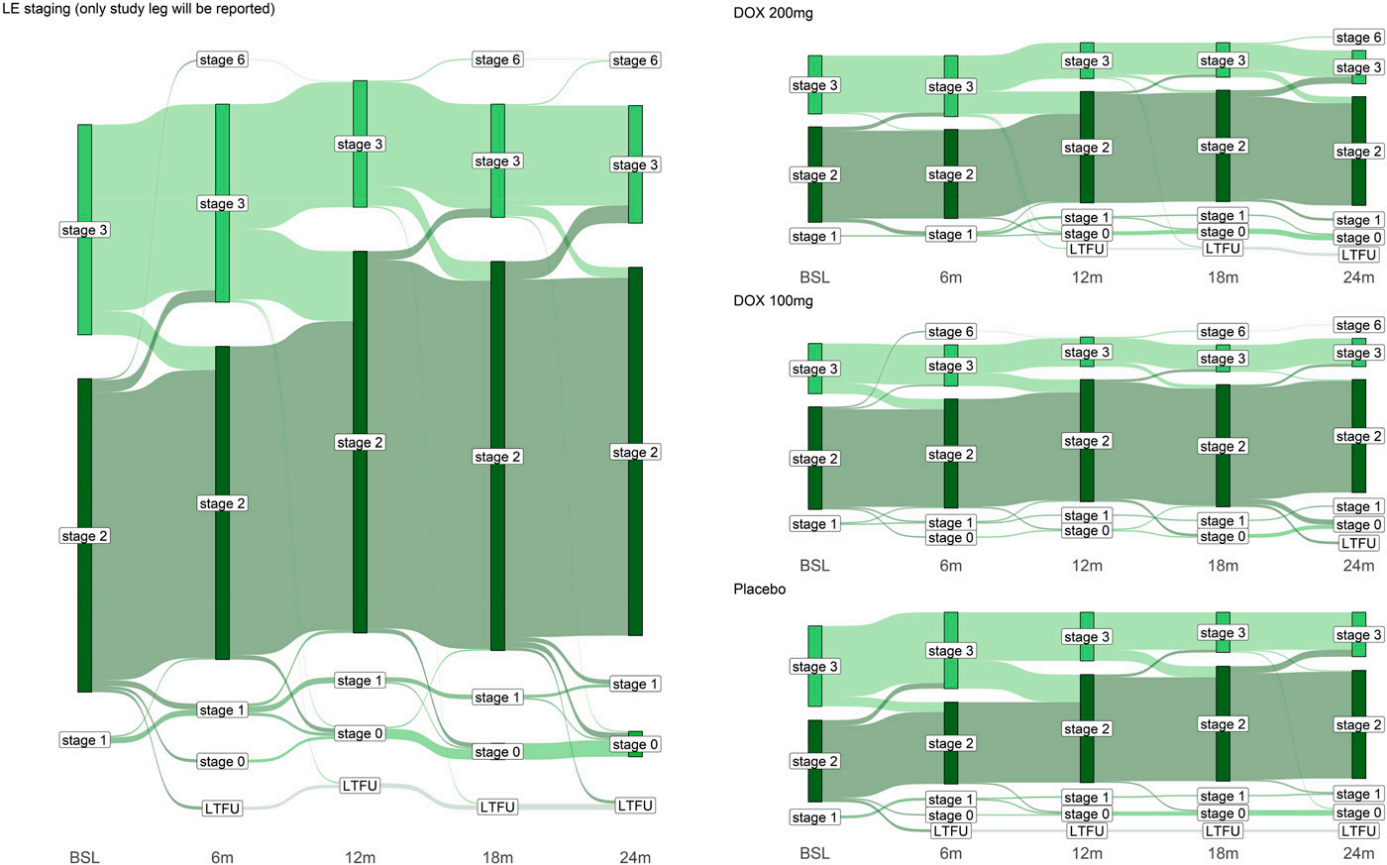
Sankey diagram of stage changes over time. The Sankey diagram shows changes in the LE stages in the ITT collective over the study period of 2 years for all participants together (left graph) and divided by the three different treatments (right graph). BSL = baseline; DOX = doxycycline; ITT = intention-to-treat; LE = lymphedema; LTFU = lost-to-follow-up.

**Figure 3.**
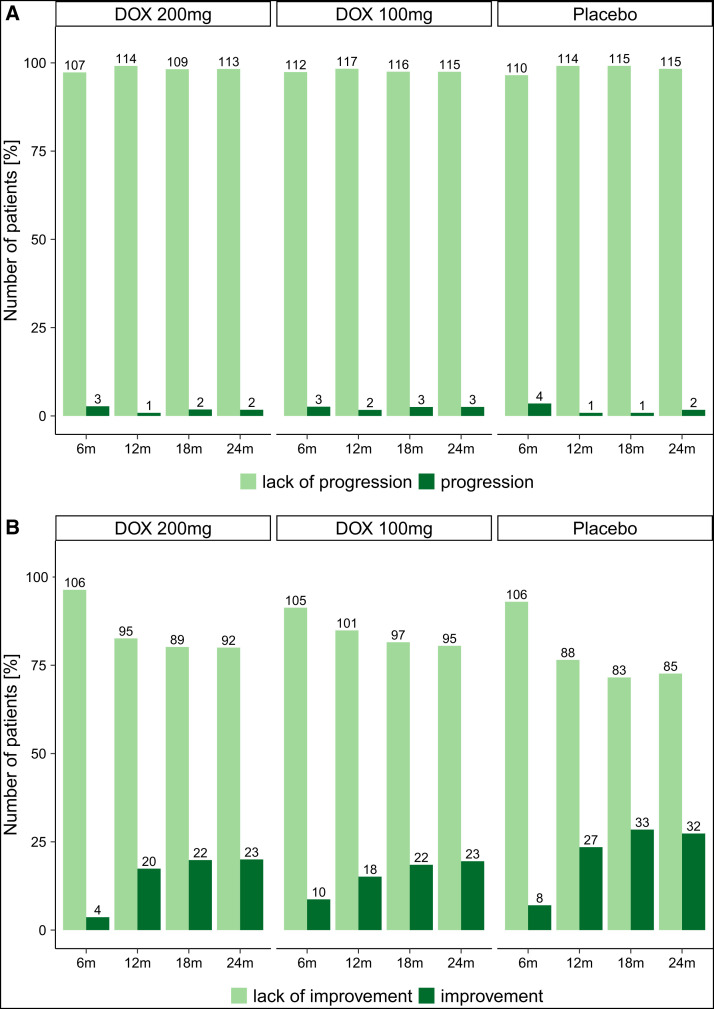

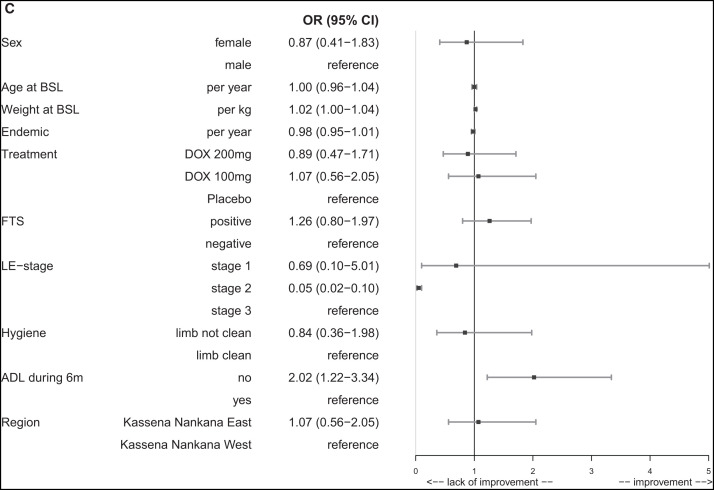
Stage progression and improvement. (**A**) The number and percentage of participants (ITT collective) who showed LE stage progression at the different follow-up time points divided by the treatment groups. (**B**) The number and percentage of participants (ITT collective) for the parameter LE stage improvement. (**C**) Forrest plot (see next page) – multivariable analysis for stage improvement over time. The Forest plot shows the variables that were included in a multivariable logistic regression model (PROC GENMOD) for the time-dependent outcome variable “improvement.” The following covariables were chosen for the model: sex (male/female), age, weight, years in endemic area, treatment (DOX 200/DOX 100/placebo), and region (Kassena Nankana East and Kassena Nankana West) as well as the following time-dependent covariables (taking changes during the follow-up period into account): LE staging (stage 1, 2 or 3), FTS positivity (active LF infection), hygiene status (limb not clean/limb clean), and ADL attack during the previous 6 months (no/yes). Odds ratios (ORs) with 95% CIs are given for each covariable. ADL = adenolymphangitis; DOX = doxycycline; FTS = filariasis test strip; ITT = intention-to-treat; LE = lymphedema; LF = lymphatic filariasis; PROC GENMOD = Procedure for Generalized Linear Models.

The Sankey diagram (Figure 2) shows the change in stage (using the Dreyer LE staging scale^10^^,^^11^) of the study legs over the study period of 24 months. *Progression* was defined as at least one stage higher at the follow-up visit than at baseline, *improvement* as at least one stage lower. In all treatment groups, a considerable proportion of patients/legs improved (at 24 months: DOX 200 mg: *n =* 23 [20%]; DOX 100 mg: *n =* 23 [19.5%]; placebo: *n =* 32 [27.4%]; Figure 3B), in contrast to only a small proportion of legs that deteriorated over the observation period (at 24 months: DOX 200 mg: *n =* 2 [1.7%]; DOX 100 mg: *n =* 3 [2.5%]; placebo: *n =* 2 [1.7%]; Figure 3A). Improvement in stage was most pronounced between 6 and 12 months for all groups, (Figures 2 and 3). A PP analysis confirmed the results of the ITT analysis (Supplemental Figure 1A and B).

**Figure 4.**
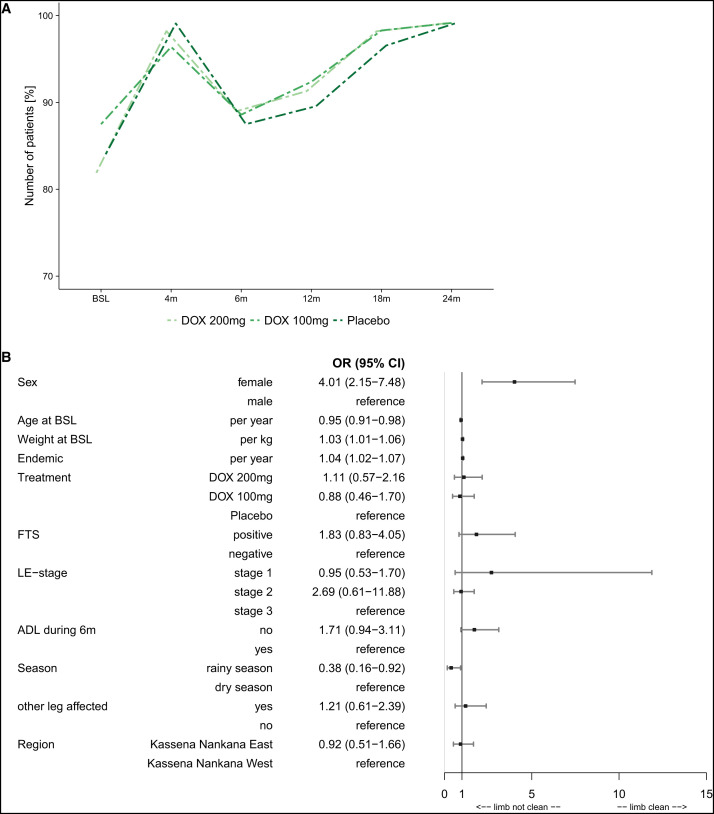
(**A**) Hygiene status – limb washed and clean. Hygiene training was carried out at baseline and at 4, 6, 12, 18, and 24 months. At the same point, participants were assessed for the hygiene of the legs. In this graph, the overall assessment “limb was washed and clean” is shown for all time points. The dotted lines indicate that not all participants were present at all time points. (**B**) Forrest plot – multivariable analysis for hygiene status over time. The Forest plot shows the variables that were included in a multivariable logistic regression model (PROC GENMOD) for the time-dependent outcome variable “limb washed and clean,” also referred to as “hygiene status.” The following covariables were chosen for the model: sex (male/female), age, weight, years in endemic area, treatment (DOX 200/DOX 100/placebo), other leg affected, and region (Kassena Nankana East and Kassena Nankana West), as well as the following time-dependent covariables (taking changes during the follow-up period into account): LE staging (stage 1, 2, or 3), ADL attack during the previous 6 months (no/yes), FTS positivity (active LF infection), and season at time of assessment (rainy or dry season). Odds ratios (ORs) with 95% CIs are given for each covariable. ADL = adenolymphangitis; BSL = baseline; DOX = doxycycline; FTS = filariasis test strip; LE = lymphedema; LF = lymphatic filariasis; PROC GENMOD = Procedure for Generalized Linear Models.

A multivariable analysis, done to identify specific factors associated with stage improvement and to account for imbalanced baseline data, confirmed that LE stage 3 was more likely to improve than LE stage 2 (OR: 0.05; *P <*0.0001; Figure 3C). Participants who did not experience an acute ADL episode during the previous 6 months were more likely to show improvement than participants who had an attack (OR: 2.02; *P =* 0.0064) (Figures 4A and 4B).

### Hygiene assessment.

There were no differences in observed hygiene status between groups, as assessed by physical examination (Figures 4A and 4B). More than 80% of trial participants had washed and clean legs at trial onset, but additional improvement was seen after enrollment, with a peak 4 months after the first training and a subsequent drop at 6 months before it continuously increased again until 24 months (Figure 4A). Factors associated with lower hygiene scores in a multivariable analysis were assessment during the rainy season (OR: 0.38; *P =* 0.0327), male sex (OR: 4.01; *P <*0.0001), increasing age (OR: 0.95; *P =* 0.0042), and lower body weight (OR: 1.03; *P =* 0.007). Longer residence in the endemic area was associated with higher hygiene scores (OR: 1.04; *P =* 0.0017). Having an ADL attack during the previous 6 months was not associated with physical examination hygiene score (OR: 1.71; *P =* 0.0789).

### Adenolymphangitis episodes.

**Figure 5.**
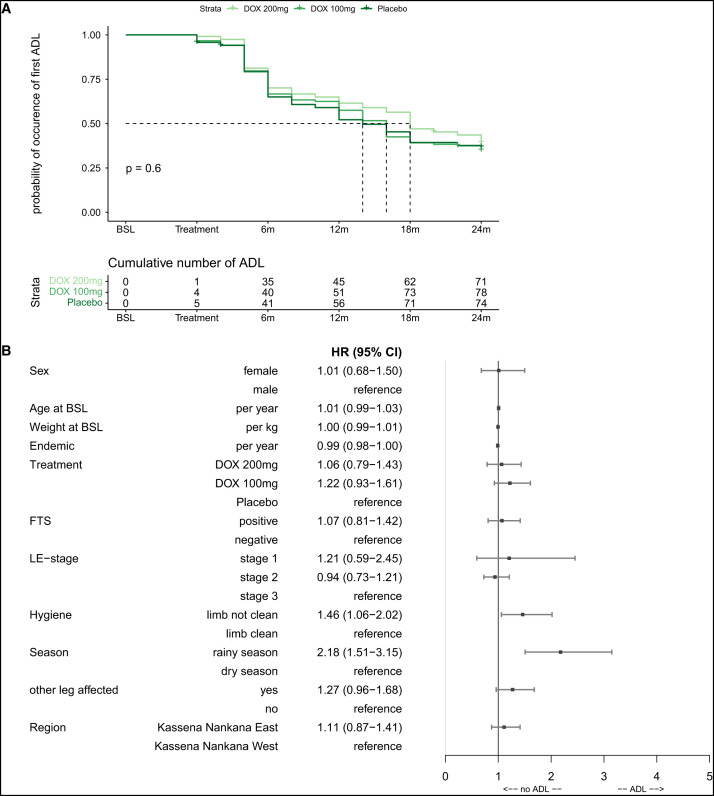
(**A**) ADL – time to first attack after treatment start. The Kaplan-Meyer curve shows the time to the occurrence of the first ADL attack after treatment onset divided by the different treatment groups. The time until 50% of the participants experienced a new attack after treatment onset was 14 months for the placebo group, 16 months for the DOX 100 group, and 18 months for the DOX 200 group. (**B**) ADL – count model – multivariable analysis on ADL attacks over time. A Cox model formulated in terms of increments in the number of events along the time line was used following an approach by Anderson and Gil^33^^,^^34^ to take not only the first occurrence of an ADL attack into account but also the fact that multiple episodes could have occurred during the study period of 2 years. The Forest plot shows the effects for the following covariables that were chosen for this model as hazard ratios (HRs) with 95% CIs: sex (male/female), age, weight, years in endemic area, treatment (DOX 200/DOX 100/placebo), other leg affected, and region (Kassena Nankana East and Kassena Nankana West), as well as the following time-dependent covariables (taking changes during the follow-up period into account): LE staging (stage 1, 2, or 3), FTS positivity (active LF infection), hygiene status (limb not clean/limb clean), and season at time of assessment (rainy or dry season). ADL = adenolymphangitis; DOX = doxycycline; FTS = filariasis test strip; LE = lymphedema; LF = lymphatic filariasis.

Time to first ADL episode was not statistically significantly different between treatment groups (Figure 5A). By 24 months, nearly 60% of all participants experienced an acute attack after start of treatment. Factors associated not only with the first ADL episode after treatment but also with the number of attacks over the 2-year study period included having legs that did not appear as clean during physical hygiene exam (HR: 1.46; *P =* 0.0209) and assessments done during the rainy season (HR: 2.18; *P ≤*0.0001).

**Figure 6.**
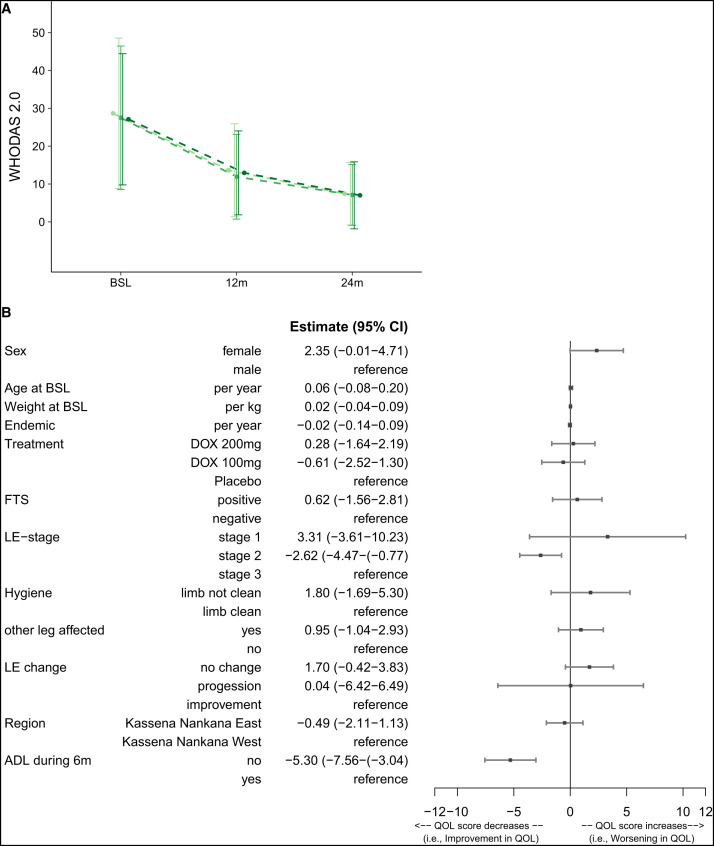
(**A**) QoL – WHODAS 2.0. The mean ± SEM of the WHODAS 2.0 score at baseline and 12 and 24 months. The dotted lines indicate that not all participants were present at all time points. (**B**) QoL – multivariable analysis for WHODAS 2.0 changes over time. To analyze the influence of covariables on the WHODAS 2.0 score, a linear mixed-effects model was used. The Forest plot shows the covariables that were used in the linear mixed-effects model, which were the following baseline covariables: sex (male/female), age, weight, years in endemic area, treatment (DOX 200/DOX 100/placebo), district (Kassena Nankana East and Kassena Nankana West), and if the other leg was also affected, as well as the following time-dependent covariables (taking changes during the follow-up period into account): LE staging (stage 1 or 2/stage 3) as well as LE change (no change/progression/improvement), FTS positivity (active LF infection, hygiene status [limb not clean/limb clean], and ADL attack during the previous 6 months (no/yes). The effects are presented with the estimate (β) and the 95% CIs. ADL = adenolymphangitis; BSL = baseline; FTS = filariasis test strip; LE = lymphedema; LF = lymphatic filariasis; QoL = quality of life; SEM = standard error of the mean; WHODAS = WHO Disability Assessment Schedule.

### Quality of life (WHODAS 2.0).

The participants in this trial started with a mean WHODAS 2.0 disability score of 27.5 ± 1.7 (SEM), which decreased to 12.8 ± 0.6 at 12 months and further to 7.2 ± 0.4 at 24 months, indicating a clear improvement in their QoL without any difference between the three treatment groups (Figure 6A).

The following main factors associated with improvement of QoL (reduced disability score) were retrieved from the multivariate linear regression model: stage 2 participants compared with stage 3 participants (estimate: −2.62; *P =* 0.0102) and participants who had no ADL attack during the previous 6 months (estimate: −5.3; *P* ≤0.0001).

### Circumference and volume measurements of legs.

The circumference and volume measurements did not lead to any relevant results or additional conclusions.

### Safety.

A total of 103 AEs were reported during treatment among 62 (17%) of the 356 participants in group A (Table 2) for the time between treatment end and 4 months after treatment onset (see Supplemental Table 4). Another 335 AEs were reported by 151 participants in the period between treatment end and the 4-month follow-up (Supplemental Table 4). Four SAEs were reported over the 24-month trial period: three deaths and one SAE in relation to a pregnancy. All three deaths were rated as unrelated to trial medication: 1) stroke in a 59-year-old man 1 month after last dose of treatment, placebo group; 2) wound infection in a 54-year-old woman 16 months after last dose of treatment, DOX 200 group; and 3) cancer in a 50-year-old woman 21 months after last dose of treatment, DOX 100 group. One woman (36 years old) from the DOX 100 group reported a macerated stillbirth of one of a pair of twins 11 months after last dose of treatment. This SAE was rated as unlikely related to the trial medication.

**Table 2:** Number of adverse events reported during treatment period

Adverse Event (MedDRA “preferred terms”)	DOX 200 mg	DOX 100 mg	Placebo	Total
Diarrhea	4 (28.6%)	6 (42.9%)	4 (28.6%)	14
Headache	5 (38.5%)	6 (46.2%)	2 (15.4%)	13
Pain	3 (23.1%)	6 (46.2%)	4 (30.8%)	13
Transaminases Increased	3 (23.1%)	5 (38.5%)	5 (38.5%)	13
Pain in Extremity	4 (33.3%)	6 (50%)	2 (16.7%)	12
Vomiting	2 (33.3%)	3 (50%)	1 (16.7%)	6
Abdominal Pain	3 (75%)	1 (25%)	0	4
Pruritus	2 (50%)	1 (25%)	1 (25%)	4
Pyrexia	2 (50%)	1 (25%)	1 (25%)	4
Arthralgia	0	2 (66.7%)	1 (33.3%)	3
Other*	7 (41.2%)	5 (29.4%)	5 (29.4%)	17
Total	35 (34%)	42 (40.8%)	26 (25.2%)	103

DOX = doxycycline.

* Other: occurrence <3 (increased blood pressure, malaise, palpitations, arthropod sting, blisters, chills, cough, eye inflammation, gangrene, infection, myalgia, nail injury, peripheral swelling, rash pruritic).

## DISCUSSION

Filarial LE of the lower limbs poses significant challenges to the social and economic livelihood of affected individuals, the majority of whom are females.^15^^,^^35^^–^^38^ The present study sought to investigate the impact of strict (and controlled) adherence to MMDP according to the Essential Package of Care recommended by the WHO, plus varying dosages of DOX in a double-blind, randomized, controlled trial. Interventions were performed in 356 study participants with early-stage filarial LE.^16^ Similar to previous studies, a significant majority of randomly recruited participants in this study were female (86.8%), underlining the disproportionate burden of lower limb LE in females in many endemic communities.^37^^–^^39^ Female participants also adhered better to hygiene measures than did males in this study, in line with findings from earlier studies showing the tendency for women to make more use of resources and support services in dealing with LE morbidity.^40^^,^^41^

The primary endpoint of this study was improvement or lack of progression in leg LE stage. Importantly, this endpoint was reached in the majority of study participants; this finding was also supported by a systematic improvement in the quality of life reported by participants across all groups over the duration of the study. In relation to leg LE improvement (measured as a reduction of at least one LE stage based on LE staging by Dreyer et al.^11^), it was observed that there was an increase in the proportion of participants across all treatment groups with improved leg LE stages. Therefore, the observed improvement in leg LE cannot be ascribed to the effect of DOX, in contrast to previous studies.^10^^,^^12^ Rather, the benefits derived by participants over the duration of this study seem to be attributable to the standard MMDP training offered to all participants, highlighting the effectiveness of hygiene-based measures alone to improve LE morbidity. Previous studies have shown that well-controlled adherence to enhanced hygiene measures is highly effective in improving LE morbidity.^42^^,^^43^ It is likely that the frequent follow-up visits and regular refresher training on standard MMDP practices in our study contributed to high adherence with hygiene practices.

A trend of general improvement in leg LE stages was seen in a proportion of study participants from all groups, with observed improvement from stage 3 to 2 across the 24-month study period. The tendency of an individual with stage 2 leg LE progressing to stage 3 was very low, whereas the reverse (improvement in LE stage from stage 3 to 2) was common for this study. A similar tendency of improvement in leg LE stages from 3 to 2 was reported in a previous DOX trial in Ghana.^10^ Interestingly, the greatest proportion of improved leg LE stages was seen between 6- and 12-month post-treatment intervention across all groups in this study. This was perhaps due to the recency of standard MMDP training offered to all study participants at baseline, with retraining done at 4, 6, 12, 18, and 24 months, in addition to the initial fervor and willingness of participants to practice these “new” MMDP or hygiene measures in hopes of seeing significant improvement in their LE conditions. Importantly, for all follow-up time points, participants who had not experienced any ADL attacks during the 6 months prior to the respective follow-up visit were twice as likely to have improvement in leg LE, again most probably as a result of the effectiveness of the standard MMDP practices described above. A prior study in Haiti reported a positive impact of systematic adherence to hygiene measures leading to significant reduction in ADL attack incidence.^39^ The importance of adherence to good hygiene was also seen in participants who had comparatively poor hygiene, as they experienced relatively more ADL attacks than participants who practiced better hygiene.

Seasonal difference in incidence of ADL attacks was another important contributory factor confirmed in this study, with more ADL attacks reported by participants in the rainy or wet season, spanning June to October, compared with the dry season, spanning November to May.^37^^,^^39^ The study participants were predominantly farmers^15^ who intensively engaged in vigorous farming activities during the rainy season and minimally undertook irrigation farming during a short defined period of the dry season. In study participant communities, the rainy season coincided with the start of treatment and the 12- and 24-month major follow-up periods, whereas the dry season coincided with the 6- and 18-month major follow-up periods. Adenolymphangitis attacks were more frequent during the rainy season, most likely owing to the cultural farming practices of study participants, which were rooted in age-old practices. According to study participants, the local culture encourages barefooted tilling and farming of land. The lack of protection for the feet or legs during such activities increases the chances of injury or entry of microorganisms into lesions on the feet or legs, which may consequently drive inflammation and ADL attack episodes in individuals with LE.^25^^,^^44^ The first ADL attack after treatment occurred earlier in the placebo group than in the DOX intervention groups. Although this was only a trend in this trial, it does support another trial from Tanzania with identical design in which the earlier occurrence of ADL in the placebo group was significant at 6 months.^45^ Perhaps, DOX may play a role in controlling opportunistic secondary bacteria that facilitate ADL attacks,^44^ hence the longer period until the next attack in the DOX intervention groups.

Another significant outcome of this study was the observation of a clear trend of improvement in the QoL of study participants across all groups. A plausible explanation for this observation may be the general adherence to hygiene measures in all groups. Interestingly, participants with leg LE stage 2 were significantly more likely to report improvement in QoL, as were participants who had not experienced any ADL attacks during the 6 months prior to the respective QoL assessment. Overall, improvement in the QoL could be attributed to the MMDP given to the patients, which in turn reduced ADL attacks. Improvement in QoL manifested as the ability of the patients to go to their farms, attend social gatherings, and do daily chores. This therefore calls for establishment of MMDP centers in endemic communities so that LE patients can access health care. It is also recommended that in countries such as Ghana, where there is a national health insurance scheme (NHIS), LE should be listed among conditions qualified for the NHIS. Furthermore, in Ghana, it would also be beneficial if LE patients could be put on Livelihood Empowerment Against Poverty, which is a social protection intervention of the Government of Ghana with the aim of reducing extreme poverty by increasing consumption and nutrition, as well as promoting access to social services and opportunities among the extremely poor and vulnerable in Ghana. This would go a long way to improve the QoL of LE patients.

Although DOX, which reduces inflammation and increases angiogenesis,^46^^–^^48^ has previously been shown to improve filarial LE and reduce LE stage irrespective of LF infection,^10^ no such benefit of DOX was observed in this study. One explanation might relate to DOX’s ability (known from animal models) to prevent new infection by incoming L3s, thereby mitigating local inflammation and angiogenesis. Because the earlier study^10^ was conducted in an area where active transmission of infection (and thus L3 intake) was ongoing^10^ whereas the present study took place in an area where transmission was almost nonexistent after several rounds of MDA,^16^ the absence of transmission (and subsequently a reduction in the burden of LF infection) and potential new infections may have obscured the previously reported ability of DOX to improve leg LE.^10^^,^^12^ In addition, because this trial did not include a control group without stringent application of the hygiene package, the effect of the hygiene measures on LE stage improvement may actually have been smaller and LE stage improvement may also have been due to less (or interrupted) transmission owing to successful MDA. In the present study, standard MMDP practices alone were seen to be broadly effective in ameliorating morbidity and improving QoL for study participants, in line with previous reports.^42^^,^^43^^,^^49^ Although it is still possible that the addition of DOX may be beneficial for treating LE in populations still experiencing active LF transmission, it is clear that for countries and communities where LF transmission has been essentially interrupted, following the WHO’s MMDP recommendations (the Essential Package of Care for LF) is the preferred approach to current patient management.

## Supplemental Materials

10.4269/ajtmh.24-0313 Supplemental Materials
