# Supplementary material for: Adherence to Hygiene Protocols and Doxycycline Therapy in Ameliorating Lymphatic Filariasis Morbidity in an Endemic Area Post-Interruption of Disease Transmission in Ghana
Source: Am J Trop Med Hyg. 2024 Oct 1;111(4 Suppl):66–82. doi: 10.4269/ajtmh.24-0313 (PMC11448491; doi:10.4269/ajtmh.24-0313)
Supplement: Supplemental Materials [file tpmd240313.SD1.pdf]

Supplementary figures 1

Figure 1a Stage progression (PP)

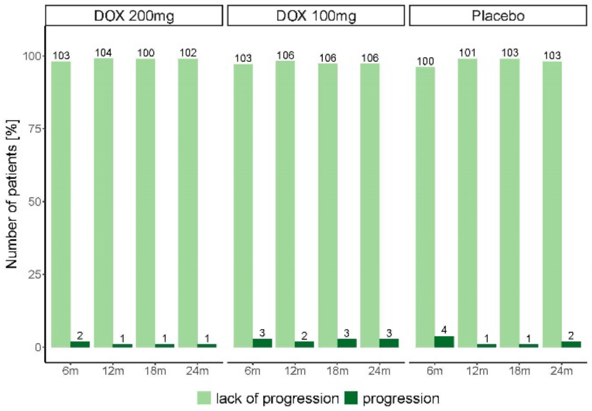

Figure 1b Stage improvement (PP)

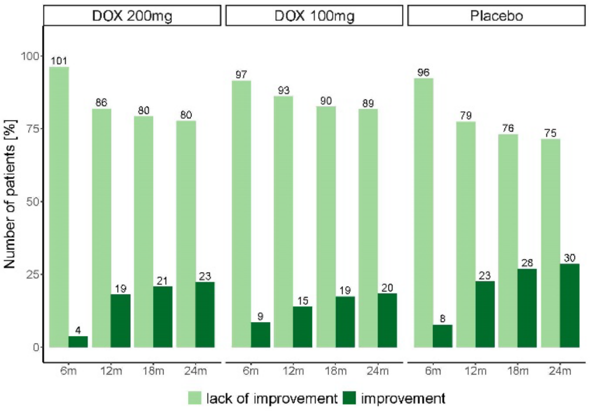

Figure 1a shows the number and percentage of participants (IPP collective) who showed LE stage progression at the different follow-up time points divided by the treatment groups. Figure 1b shows the same for the parameter LE stage improvement.

Supplementary table 1: List of study communities in Kassena Nankana East Municipal (KNEM)

Kassena Nankana East Municipal (KNEM)						
**Sub-districts**						
	**Pungu**	**Wuru**	**Manyoro**	**Navrongo East**	**Vanania/ Kapania**	**Biu/Sensa/Kologo**
**Communities**	1. Lower Talania<br>2. Upper Talania<br>3. Machoro<br>4. Bavegnia<br>5. Bawiu A&B<br>6. Wusugu<br>7. Punyoro<br>8. Yitonia<br>9. Tekuru<br>10. Dembasenia<br>11. Badayoro	1. Gia-Bngania<br>2. Gia-Jammagisongo<br>3. Gia Kwosongo<br>4. Gia- Nangwao<br>5. Bonia 1 & 2<br>6. Lower Nangalikinia<br>7. Upper Nangalikinia<br>8. Wuru Central<br>9. Korania	1. Wanjania<br>2. Sakonia<br>3. Akumbisi<br>4. Saabisi<br>5. Dambisi<br>6. Gumongu<br>7. Gerebisi<br>8. Natugnia-Dazongo<br>9. Nyogsi<br>10. Gware/ Chilla	1. Abepingo A & B<br>2. Akukongo<br>3. Gayingo-Doba<br>4. Akuka<br>5. Akurugu-Dabo<br>6. Agurum-aluku<br>7. Korigo-Kandango<br>8. Gingabnia<br>9. Badania	1. Janania<br>2. Vunania<br>3. Gaani<br>4. Kapania<br>5. Tampola	1. Sensa<br>2. Kologo Zou<br>3. Kologo Tua<br>4. Kologo Tindango

Supplementary table 2: List of study communities in Kassena Nankana West District (KNW)

**Kassena Nankana West (KNW)**							
**Sub-districts**							
**Communities**	**Mirigu**	**Sirigu**	**Kandiga**	**Chiana**	**Paga**	**Navio**	**Nakolo**
	1. Chengo<br>2. Nayire<br>3. Kassalingo<br>4. Tikongo<br>5. Abugzidone<br>6. Gonum<br>7. Gayingo<br>8. Kumbusingo<br>9. Pungubisi<br>10. Alembilla<br>11. Kansaam<br>12. Woligum<br>13. Azinzorka<br>14. Aburuwasah<br>15. Nyongo<br>16. Zampengo<br>17. Dorsum	1. Wugingo<br>2. Abillateo<br>3. Dazongo<br>4. Puwellingo<br>5. Tangasia<br>6. Tidongo<br>7. Bugsongo<br>8. Basengo-Moo<br>9. Nyango-lingo<br>10. Basengo-Azuo-Done<br>11. Amutanga	1. Azeadoma<br>2. Agandaa<br>3. Azuredone<br>4. Tindongo<br>5. Nindongo<br>6. Kurugu<br>7. Akamo<br>8. Atiyorum<br>9. Kaase<br>10. Akamo	1. Gwernia<br>2. Gaania<br>3. Nyangania<br>4. KalvioAbulu<br>5. Kanania<br>6. Kalvio-Aboenia<br>7. Gugoro<br>8. Saboro<br>9. Adognia<br>10. Wurunia<br>11. Yidania<br>12. Asunia<br>13. Kafania	1. Kakungu North<br>2. Chaina<br>3. Batiu<br>4. Bisao<br>5. Baloo<br>6. Kajello 1 & 2<br>7. Nania<br>8. Zenga 1 & 2<br>9. Nabio<br>10. Babile<br>11. Gwaru 1&2	1. Bagtua<br>2. Kasili<br>3. Navio Tazika<br>4. Kazugu<br>5. Badunu<br>6. Sakaa<br>7. Tigakuri<br>8. Sanwo	1. Nakolo Central<br>2. Banyono<br>3. Kuliya<br>4. Nakolo Tazika<br>5. Boania<br>6. Buburu-yuu<br>7. Bugani

Supplementary table 3a Additional baseline data – demographics

			DOX 200mg	DOX 100mg	Placebo	Total	*p-value*
**Marital Status**	Never married	N (%)	1 (0.9%)	2 (1.7%)	8 (6.7%)	11 (3.1%)	
	Currently married	N (%)	67 (57.3%)	72 (60%)	82 (68.9%)	221 (62.1%)	
	Separated	N (%)	1 (0.9%)	2 (1.7%)	0 (0%)	3 (0.8%)	
	Divorced	N (%)	5 (4.3%)	3 (2.5%)	2 (1.7%)	10 (2.8%)	
	Widowed	N (%)	43 (36.8%)	41 (34.2%)	27 (22.7%)	111 (31.2%)	***0.0264[a]***
**Married**	no	N (%)	50 (42.7%)	48 (40%)	37 (31.1%)	135 (37.9%)	
	yes	N (%)	67 (57.3%)	72 (60%)	82 (68.9%)	221 (62.1%)	*0.1557[a]*
**Main work status**	Paid work	N (%)	1 (0.9%)	2 (1.7%)	3 (2.5%)	6 (1.7%)	
	Self-employed	N (%)	115 (98.3%)	113 (94.2%)	113 (95%)	341 (95.8%)	
	Non-paid work	N (%)	0 (0%)	1 (0.8%)	0 (0%)	1 (0.3%)	
	Student	N (%)	0 (0%)	0 (0%)	2 (1.7%)	2 (0.6%)	
	Keeping house/ homemaker	N (%)	0 (0%)	1 (0.8%)	0 (0%)	1 (0.3%)	
	Retired	N (%)	1 (0.9%)	1 (0.8%)	0 (0%)	2 (0.6%)	
	Unemployed (health reason)	N (%)	0 (0%)	1 (0.8%)	0 (0%)	1 (0.3%)	
	Unemployed (other reasons)	N (%)	0 (0%)	1 (0.8%)	1 (0.8%)	2 (0.6%)	*0.7293[a]*

[a] Fishers exact test

Supplementary table 3b Additional baseline data - Participants characteristics

			DOX 200mg	DOX 100mg	Placebo	Total	*p-value*
**LE staging right leg**	0 - No abnormality	N (%)	42 (35.9%)	44 (36.7%)	47 (39.5%)	133 (37.4%)	
	1 - Swelling is reversible	N (%)	1 (0.9%)	2 (1.7%)	2 (1.7%)	5 (1.4%)	
	2 - Swelling is not reversible	N (%)	42 (35.9%)	46 (38.3%)	35 (29.4%)	123 (34.6%)	
	3 - Presence of shallow skin folds	N (%)	31 (26.5%)	27 (22.5%)	32 (26.9%)	90 (25.3%)	
	5 - Presence of deep skin folds	N (%)	0 (0%)	0 (0%)	1 (0.8%)	1 (0.3%)	
	6 - Presence of mossy lesions	N (%)	1 (0.9%)	1 (0.8%)	2 (1.7%)	4 (1.1%)	*n.d.*
**LE staging Left leg**	0 - No abnormality	N (%)	49 (41.9%)	49 (40.8%)	51 (42.9%)	149 (41.9%)	
	1 - Swelling is reversible	N (%)	2 (1.7%)	1 (0.8%)	2 (1.7%)	5 (1.4%)	
	2 - Swelling is not reversible	N (%)	43 (36.8%)	51 (42.5%)	32 (26.9%)	126 (35.4%)	
	3 - Presence of shallow skin folds	N (%)	23 (19.7%)	17 (14.2%)	34 (28.6%)	74 (20.8%)	
	6 - Presence of mossy lesions	N (%)	0 (0%)	2 (1.7%)	0 (0%)	2 (0.6%)	*n.d.*
**Last ADL attack[e] (months before V1)**		N	112	119	118	349	
		median, iqr	14; 11	14; 12	15; 12	14; 12	
		95% CI median	[14; 15]	[14; 15]	[14; 15]	[14; 15]	
		min - max	2-89	2-76	2-64	2-89	*0.4815*[c]
**Number of attacks within the last year (all patients)**		N	101	103	108	312	
		median, iqr	1; 1	1; 1	1; 1	1; 1	
		95% CI median	[1; 1]	[1; 1]	[1; 1]	[1; 1]	
		min - max	0-6	0-6	0-9	0-9	*0.9847*[c]
**Duration of attacks (average within the last year (days)) (all patients)**		N	114	119	118	351	
		median, iqr	5; 7	5; 5	5; 4	5; 5	
		95% CI median	[4; 7]	[4; 7]	[4; 7]	[5; 6]	
		min - max	0-90	0-30	0-90	0-90	*0.5065*[c]
**Duration of attacks (average within the last year (days)) (only patients who had attacks)**		N	85	90	98	273	
		median, iqr	7; 4	7; 6	7; 5	7; 5	
		95% CI median	[5; 7]	[6; 7]	[5; 7]	[7; 7]	
		min - max	1-90	2-30	1-90	1-90	*0.9906*[c]
**Observer Assessed Cleanliness Score[f]**		N	117	120	119	356	
		median, iqr	1; 0.2	1; 0.2	1; 0.3	1; 0.3	
		95% CI median	[0.8; 1]	[1; 1]	[0.9; 1]	[1; 1]	
		min - max	0.3-1	0.3-1	0.3-1	0.3-1	*0.6799*[c]

Continuation of supplementary table 3b Additional baseline data - Participants characteristics

		DOX 200mg	DOX 100mg	Placebo	Total	*p-value*
**Observer Assessed Skin Score[f]**	N	117	120	119	356	
	median, iqr	0.9; 0.2	0.9; 0.2	0.9; 0.2	0.9; 0.2	
	95% CI median	[0.9; 0.9]	[0.9; 0.9]	[0.8; 0.9]	[0.9; 0.9]	
	min - max	0.7-1	0.6-1	0.4-1	0.4-1	*0.4262[c]*
**Self Assessed Hygiene Score[f]**	N	103	101	102	306	
	median, iqr	0.57; 0.05	0.57; 0.05	0.57; 0.05	0.57; 0.05	
	95% CI median	[0.57; 0.57]	[0.57; 0.62]	[0.57; 0.57]	[0.57; 0.57]	
	min - max	0.52-0.81	0.52-0.90	0.43-0.76	0.43-0.90	***0.0133[c]***
**WHODAS 2.0 Physical LF Sub-score (Items S1, S7-S9)**	N	117	120	119	356	
	median, iqr	8.3; 10.4	6.3; 9.4	6.3; 8.3	6.3; 9.4	
	95% CI median	[6.25; 8.34]	[6.25; 8.34]	[6.25; 8.34]	[6.25; 8.34]	
	min - max	0-33.3	0-31.2	0-25	0-33.3	*0.6116[c]*
**WHODAS 2.0 Not Physical LF Sub-Score (Items S2-S6, S10-S12)**	N	117	120	119	356	
	median, iqr	16.7; 16.7	14.6; 18.8	14.6; 16.7	14.6; 18.8	
	95% CI median	[12.5; 20.8]	[12.5; 16.7]	[12.5; 18.7]	[12.5; 16.7]	
	min - max	0-66.6	0-52.1	0-54.2	0-66.6	*0.88[c]*
**H1: Overall, in the past 30 days, how many days were these difficulties present?**	N	117	120	119	356	
	median, iqr	21; 26	24; 25	30; 24	22.5; 25	
	95% CI median	[11; 30]	[16; 30]	[14; 30]	[16; 30]	
	min - max	0-30	0-30	0-30	0-30	*0.8318[c]*
**H2: In the past 30 days, for how many days were you totally unable to carry out your usual activities or work because of any health condition?**	N	117	120	119	356	
	median, iqr	0; 3	0; 3	0; 2	0; 2	
	95% CI median	[0; 0]	[0; 0]	[0; 0]	[0; 0]	
	min - max	0-15	0-14	0-14	0-15	*0.4095[c]*
**H3: In the past 30 days, not counting the days that you were totally unable, for how many days did you cut back or reduce your usual activities or work because of any health condition?**	N	117	120	119	356	
	median, iqr	21; 27	18; 27	20; 26	20; 27	
	95% CI median	[7; 27]	[14; 27]	[10; 30]	[14; 26]	
	min - max	0-30	0-30	0-30	0-30	*0.7443[c]*

[a] Fishers exact test [b] ANOVA [c] Kruskal-Wallis test [d] Chi-square test

[e] For some participants it was very difficult to recall the history of ADL attacks. Therefore, some questions were not answered.

[f] For "overall hygiene" did the assessor evaluate whether the legs were generally well washed and cared for. To answer this question, the assessor had several other questions available within the questionnaire, which have been summarized as scores in this table.

Supplementary table 4 AEs reported between treatment end and 4 month follow-up

Adverse event (MedDRA "Preferred Terms")	DOX 200	DOX 100	Placebo	Total
**Headache**	23 (42.6%)	21 (38.9%)	10 (18.5)	54
**Pain**	16 (31.4%)	18 (35.3%)	17 (33.3%)	51
**Malaria**	17 (43.6%)	9 (23.1%)	13 (33.3%)	39
**Pain in extremity**	10 (26.3%)	9 (23.7%)	19 (50%)	38
**Back pain**	14 (58.3%)	3 (12.5%)	7 (29.2%)	24
**Diarrhoea**	8 (40%)	5 (25%)	7 (35%)	20
**Abdominal discomfort/pain**	9 (60%)	2 (13.3%)	4 (26.7%)	15
**Arthralgia**	7 (63.6%)	1 (9.1%)	3 (27.3%)	11
**Cough**	4 (36.4%)	6 (64.5%)	1 (9.1%)	11
**Chest pain**	3 (37.5%)	3 (37.5%)	2 (25%)	8
**Hypertension**	2 (33.3%)	3 (50%)	1 (16.7%)	6
**Influenza**	2 (33.3%)	1 (16.7%)	3 (50%)	6
**Pruritus**	2 (40%)	2 (40%)	1 (20%)	5
**Dizziness**	2 (50%)	1 (25%)	1 (25%)	4
**Gastroenteritis**	2 (50%)	2 (50%)	0	4
**Pyrexia**	2 (66.7%)	0	1 (33.3%)	3
**Other***	12 (33.3%)	13 (36.1%)	11 (30.6%)	36
**Total**	135 (40.3%)	99 (29.6%)	101 (30.1%)	335

*Other – occurrence < 3 (Candida infection, Catarrh, Decreased appetite, Eye pain, Gastritis, Peripheral swelling, Skin ulcer, Asthenia, Carbuncle, Cerebrovascular accident, Chills, Contusion, Face injury, Flank pain, Furuncle, Hypoaesthesia, Infection, Limb injury, Lymphangitis, Migraine, Muscle spasms, Musculoskeletal stiffness, Pain in jaw, Rash, Stress, Toothache, Upper limb fracture, Varicella, Wound)
